# Maximizing Average Throughput of Cooperative Cognitive Radio Networks Based on Energy Harvesting

**DOI:** 10.3390/s22228921

**Published:** 2022-11-18

**Authors:** Yaqing Wang, Shiyong Chen, Yucheng Wu, Chengxin Zhao

**Affiliations:** School of Microelectronics and Communication Engineering, Chongqing University, Chongqing 400044, China

**Keywords:** network average throughput, RF energy harvesting, cooperative cognitive radio network, resource allocation

## Abstract

Energy harvesting (EH) and cooperative communication techniques have been widely used in cognitive radio networks. However, most studies on throughput in energy-harvesting cooperative cognitive radio networks (EH-CCRNs) are end-to-end, which ignores the overall working state of the network. For the above problems, under the premise of prioritizing the communication quality of short-range users, this paper focuses on the optimization of the EH-CCRN average throughput, with energy and transmission power as constraints. The formulated problem was an unsolved non-deterministic polynomial-time hardness (NP-hard) problem. To make it tractable to solve, a multi-user time-power resource allocation algorithm (M-TPRA) is proposed, which is based on sub-gradient descent and unary linear optimization methods. Simulation results show that the M-TPRA algorithm can improve the average throughput of the network. In addition, the energy consumed by executing the M-TPRA algorithm is analyzed.

## 1. Introduction

In recent years, with the rapid growth of the number of mobile users and wireless communication devices, the demand for spectrum resources has increased dramatically. However, studies have shown that the low utilization of the allocated spectrum exacerbates the lack of spectrum resources [1]. The cognitive radio (CR) technology proposed by Mitola allows the secondary user (SU) to opportunistically use the spectrum of the primary user (PU) under the premise of ensuring the communication quality of PU [2]. The method improves the spectrum utilization and alleviates the problem of the shortage of spectrum resources [3,4]. Meanwhile, EH and cooperative communication technologies are considered to be the key technologies to improve the throughput of various wireless networks, including the cognitive radio network (CRN).

EH technology is utilized to solve the problem where key functions in CR technology (such as spectrum sensing and spectrum prediction) increase the energy consumption of equipment. Attempts have been made to reduce the overall energy consumption of the devices by energy-saving technology [5,6], but most CRNs are composed of battery-powered wireless devices, and energy-saving technology alone cannot fundamentally solve the problem of energy consumption. EH is a technology that provides a new way of powering the network by extracting energy from sources including solar [7], wind [8], radio frequency (RF) [9] and other energy sources. Among them, RF energy has become meaningful because of its reliability and sustainability. Wireless power transmission (WPT) technology derived from EH is widely used. In ref. [10], WPT duration, transmission time allocation of each edge device and the partial offloading decision are jointly optimized in order to maximize the sum computation rate. An online offloading algorithm based on deep reinforcement learning (DRL) is designed to solve the optimization problem. Zheng et al. studied the WPT-aided cell-free massive MIMO system. The harvested energy (HE) from the downlink WPT is used to support both uplink data and pilot transmission, which improves the spectral efficiency of the system [11]. In addition, the application of EH technology in CRNs greatly improves the performance of the CRN network. In the research of EH technology in CRNs (EH-CRN), the existing works mainly focus on two aspects: outage probability [12,13] and throughput [14,15,16,17]. An algorithm based on Proximal Policy Optimization (PPO) was proposed to maximize the network throughput of SU, with energy and Quality of Service (QoS) constraints [14]. In ref. [15], energy and collision were taken into account to maximize the average network throughput. Talukdar et al. analyzed the influence of multiple parameters on the network throughput of EH-CRN [16]. Liu et al. maximized the achievable throughput of EH-CRN by deriving the optimal decision threshold in the cooperative spectrum strategy [17].

Cooperative communication technology can effectively improve the throughput of the cognitive network while expanding the coverage of the network [18]. Cooperative cognitive radio networks (CCRNs) are a new type of network that combine cognitive radio technology and cooperative communication technology [19]. In CCRNs, SU forwards the information of PU in a cooperative relay manner and, in return, grants a part of the channel access time. Information cooperation in CCRNs can reduce outage probability [20,21] and improve network throughput [22,23]. In ref. [22], the end-to-end throughput was improved by optimizing the hop-by-hop relay selection strategy. An asymmetric resource allocation scheme with asymmetric transmission duration was proposed, and simulation verified that the scheme improved the system throughput [23].

Compared with CRN, although the network performance of CCRNs have greatly improved, they also face the problem of high energy consumption. Since the performance of EH-CRN throughput does not consider the situation of cooperative communication [14,15,16,17], the combination of CCRNs and EH-CRNs has great practical significance for future wireless communication technology research [24,25,26,27]. The simultaneous wireless information and power transfer (SWIPT) transmission concept first proposed by Varshney provided a theoretical basis for the combination of EH-CRN and CCRN [24]. By optimizing the transmission power and time switching factor of the SU for data transmission and EH, ref. [25] maximized the long-term throughput of the secondary user network. A relay selection optimization strategy was adopted to improve the throughput of the PU network [26]. The impact of the number of users on the overall network throughput was analyzed in the joint cooperative mode [27]. However, the optimization goals of refs. [25,26] are end-to-end throughput, which cannot reflect the overall working state of the cognitive network. Although the maximum total network throughput is improved in ref. [27], it ignores the effects of other parameters, such as transmit power.

This work explores the SWIPT model in the context of a multi-user EH-CCRN with the goal of maximizing the average network throughput. The system contains a pair of PUs and a pair of SUs. Secondary user transmitter (ST) does not have the original battery and can only collect energy from the RF signal of PU. At the same time, ST uses the RF energy to forward the information of PU. In addition, the CRN adopts the underlay access strategy and the decode-and-forward relay strategy. In this paper, the transmit power of PU and SU is limited to an acceptable range. A more practical cooperative transmission scenario is considered. The main contributions of the paper are as follows:

(1) A time-power joint optimization model with the goal of maximizing the network’s throughput is proposed and analyzed. The optimization model is constrained by transmission power, energy and interruption. Moreover, we comprehensively analyze the impacts of different key parameters on the average throughput of EH-CCRNs, i.e., the transmission power of PU, the system time switching factor and distance, etc.
(2) A power splitting factor expression at SU is proposed, on the basis that the effect of time switching factor on the network average throughput is independent of this factor. We provide a detailed analysis of the influence of this factor on the communication quality of short-range users.
(3) A multi-user time-power resource allocation algorithm (M-TPRA) is proposed. Firstly, M-TPRA transforms non-convex optimization problems into convex optimization problems by introducing slack variables. Secondly, using the idea of hierarchical optimization, the optimization problem is divided into two sub-problems: power control and time allocation. Thirdly, the power control is obtained by sub-gradient descent, and time allocation is obtained by unary linear optimization. Finally, we analyze the energy consumed by implementing the M-TPRA algorithm.

The rest of this paper is organized as follows. In Section 2, we introduce the system model and problem description. In Section 3, we present a solution to the time-power optimization problem. Section 4 provides the simulation results. Finally, a summary of the work is given in Section 5.

## 2. System Model

**Figure 1:**
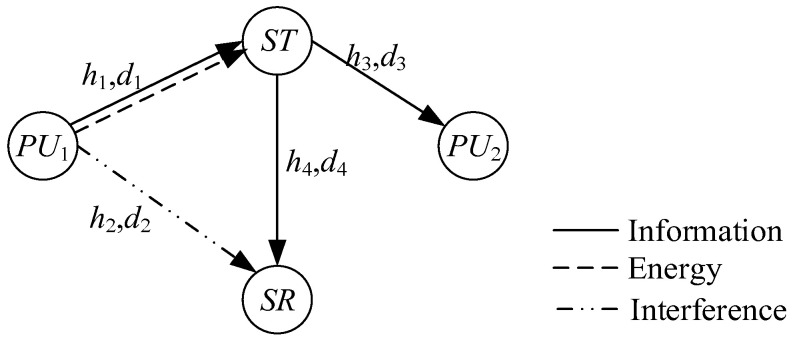
System model.

Figure 1 illustrates an underlay EH-CCRN which consists of a primary user transmitter ($PU_{1}$), primary user receiver ($PU_{2}$), ST and secondary user receiver (SR). We assume that there is no direct communication link between $PU_{1}$ and $PU_{2}$ due to distance and shadow fading. As a relay, ST can collect energy from the RF signal of $PU_{1}$ and obtain some resources (time, spectrum) to decode-forward information. In addition, all channels are independent and identically distributed and subject to Rayleigh fading. The channel coefficients remain unchanged for block time T. The notation list in Appendix A summarizes the following main variables and parameters used in this study.

**Figure 2:**
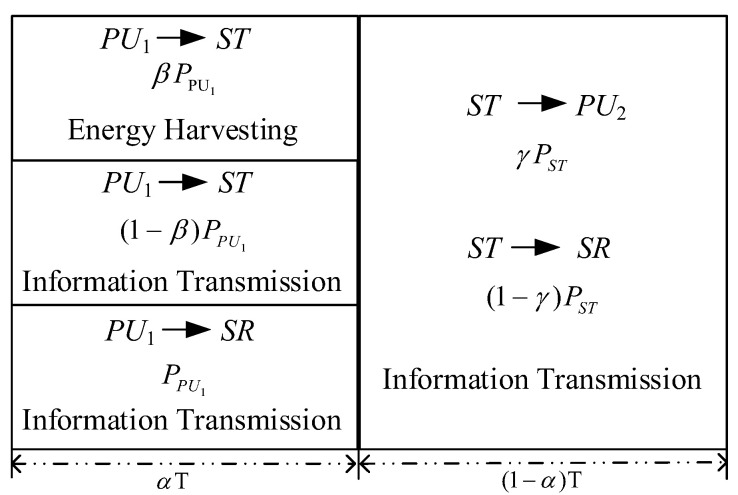
System transmission protocol.

The system transmission protocol is divided into two stages, as shown in Figure 2. In the first stage, $PU_{1}$ broadcasts, and ST uses the power splitting (PS) receiving scheme for EH and information decoding (ID). In the second stage, ST uses the harvested energy to transmit information to SR and $PU_{2}$.

### 2.1. $PU_{1}$ Broadcasting

$PU_{1}$ broadcasts signal $s_{PU1}$, and the signal received by ST is given by

(1) $$y_{ST}=\sqrt{\frac{P_{PU_{1}}}{d_{1}^{m}}}h_{1}s_{PU_{1}}+n_{ST}$$

where $P_{PU1}$ is the transmission power of $PU_{1}$, m is path loss index, $h_{1}$ is the channel coefficient between $PU_{1}$ and ST, $d_{1}$ is the distance between $PU_{1}$ and ST, and $n_{ST}$ is zero-mean additive white Gaussian noise at ST with variance $\sigma_{ST}^{2}$.

Thus, the signal-to-noise ratio (SNR) at ST is described as

(2) $${\mathrm{SNR}}_{ST}=\frac{(1-\beta )P_{PU_{1}}{\left|h_{1}\right|}^{2}}{d_{1}^{m}\sigma_{ST}^{2}}$$

According to the Shannon Theorem, the maximum transmission rate between $PU_{1}$ and ST can be expressed as

(3) $$v_{1}=\alpha \cdot \log_{2}(1+\frac{(1-\beta )P_{PU_{1}}h_{1}^{2}}{d_{1}^{m}\sigma_{ST}^{2}})$$

Furthermore, the energy $E_{ST}$ collected by ST can be written as

(4) $$E_{ST}=\frac{\alpha \beta \eta {\left|h_{1}\right|}^{2}P_{PU_{1}}T}{d_{1}^{m}}$$

where α (0<α<1) is the time switching factor, β (0<β<1) is the power-splitting factor at $PU_{1}$ and η (0<η<1) is the energy conversion efficiency.

In particular, since the $PU_{1}$ transmits information by broadcasting, it will cause certain interference to the SR. Hence, the interference signal received by SR is

(5) $$y_{SR}=\sqrt{\frac{p_{PU_{1}}}{d_{2}^{m}}}h_{2}s_{PU_{1}}+n_{SR}$$

where $h_{2}$ is the channel coefficient between $PU_{1}$ and SR, $d_{2}$ is the distance between $PU_{1}$ and SR, and $n_{SR}$ is zero-mean additive white Gaussian noise at SR with variance $\sigma_{SR}^{2}$. We assume that the SR can successfully decode the received signal transmitted by the ST in the second stage, which can effectively eliminate the interference of $PU_{1}$ [28].

### 2.2. ST Broadcasting

After ST decodes the signal transmitted by $PU_{1}$ successfully, it broadcasts the signal $s_{ST}$ with the transmission power $P_{ST}$. ST divides $P_{ST}$ into two parts: $\gamma P_{ST}$ and $(1-\gamma )P_{ST}$, where γ (0<γ<1) is the power-splitting factor at ST, $\gamma P_{ST}$ is the power of the signal $s_{PU2}$ sent to $PU_{2}$, and $(1-\gamma )P_{ST}$ is the power of the signal $s_{SR}$ sent to SR. The broadcast signal of ST can be expressed as

(6) $$s_{ST}=\sqrt{\gamma P_{ST}}s_{PU_{2}}+\sqrt{(1-\gamma )P_{ST}}s_{SR}$$

On the one hand, the signal received by $PU_{2}$ is

(7) $$y_{PU_{2}}=\sqrt{\frac{\gamma P_{ST}}{d_{3}^{m}}}h_{3}s_{ST}+n_{PU_{2}}$$

where $h_{3}$ is the channel coefficient between $PU_{2}$ and ST, $d_{3}$ is the distance between $PU_{2}$ and ST, $n_{PU2}$ is zero-mean additive white Gaussian noise at $PU_{2}$ with variance $\sigma_{PU2}^{2}$. Signal-to-interference-plus-noise ratio (SINR) at $PU_{2}$ can be expressed as

(8) $${\mathrm{SIN}R}_{PU_{2}}=\frac{\gamma P_{ST}{\left|h_{3}\right|}^{2}}{d_{3}^{m}\sigma_{PU_{2}}^{2}}$$

The maximum transmission rate on the channel between ST and $PU_{2}$ can be written as

(9) $$v_{2}=(1-\alpha )\cdot \log_{2}(1+\frac{\gamma P_{ST}h_{3}^{2}}{d_{3}^{m}\sigma_{PU_{2}}^{2}})$$

On the other hand, the signal received by SR after eliminating the interference is

(10) $$y_{SR}=\sqrt{\frac{(1-\gamma )P_{ST}}{d_{4}^{m}}}h_{4}s_{2}+n_{SR}$$

Therefore, SINR at SR is described as

(11) $${\mathrm{SIN}R}_{SR}=\frac{(1-\gamma )P_{ST}{\left|h_{4}\right|}^{2}}{d_{4}^{m}\sigma_{SR}^{2}}$$

On the channel between ST and SR, the maximum transmission rate can be expressed as

(12) $$v_{3}=(1-\alpha )\cdot \log_{2}(1+\frac{(1-\gamma )P_{ST}h_{4}^{2}}{d_{4}^{m}\sigma_{SR}^{2}})$$

### 2.3. Problem Formulation

In EH-CCRNs, when $v_{1},v_{2}$ and $v_{3}$ are lower than the target rate $R_{t}$, the link is interrupted. Assuming that the system is not interrupted within the time T, $v_{1},v_{2}$ and $v_{3}$ must satisfy the following:

(13) $$\begin{array}{l} C1\text{:}\;v_{1}>R_{t}\\ C2\text{:}\;v_{2}>R_{t}\\ C3\text{:}\;v_{3}>R_{t} \end{array}$$

Assuming that the SU does not have battery and can only use the capacitor for energy storage, the energy required by SU in the second stage can only come from the collection in the first stage, so $E_{ST}$ must satisfy the following:

(14) $$C4\text{:}\;E_{ST}\geq (1-\alpha )T*P_{ST}$$

According to the characteristics of the CRN, $P_{ST}$ is constrained by the interference threshold $I_{th}$, namely:

(15) $$C5\text{:}\;P_{ST}\leq I_{th}$$

To prevent interference with public systems, $P_{PU_{1}}$ should be constrained by Maximum transmission power $P_{T\max}$, namely

(16) $$C6\text{:}\;P_{PU_{1}}\leq P_{T\max}$$

The average network throughput τ is

(17) $$\begin{array}{ll} \tau & =\frac{1}{T}(v_{1}T+v_{2}T+v_{3}T)\\ & =v_{1}+v_{2}+v_{3} \end{array}$$

The research goal in this paper is to maximize average throughput of the network while ensuring the quality of user communication. Therefore, the throughput optimization problem can be described as

(18) $$\begin{array}{lll} & P1: & \underset{P_{PU_{1}},\alpha ,\gamma }{\max}v_{1}+v_{2}+v_{3}\\ s.t. & & C1,C2,C3,\;C4,C5,C6,\\ & & C7:\;0<\alpha <1,0<\beta <1,0<\gamma <1,\\ & & C8:\;P_{PU_{1}}>0,P_{ST}>0 \end{array}$$

Equation (18) is the optimizing problem. The optimization variables are the transmission power $P_{PU1}$, the time switching factor α, and the power splitting factor γ at ST. C1~C8 are the constraints of the optimizing problem. C7 and C8 are constraints on the value range of α, β, γ, making the system model more realistic.

## 3. Multi-User Time-Power Resource Allocation Algorithm (M-TPRA)

Equation (18) is a non-convex problem (Appendix B for the proof), and it is difficult to directly obtain the solution. Therefore, it is necessary to make it a convex optimization.

In the model proposed in this paper, we assume that the effect of α on the network average throughput is independent of γ. That is, the network average throughput τ is independent of γ after taking the derivative of α:

(19) $$\;\frac{\partial \tau^{2}}{\partial \alpha \partial \gamma }=0$$

Taking the expression of $\tau =v_{1}+v_{2}+v_{3}$ into Equation (19), we can obtain

(20) $$\frac{P_{ST}h_{3}^{2}}{d_{3}^{m}\sigma_{PU_{2}}^{2}+\gamma P_{ST}h_{3}^{2}}=\frac{P_{ST}h_{4}^{2}}{d_{4}^{m}\sigma_{SR}^{2}+(1-\gamma )P_{ST}h_{4}^{2}}$$

From Equation (20), $\gamma^{*}$ can be solved and expressed as

(21) $$\gamma^{*}=\frac{h_{3}^{2}d_{4}^{m}\sigma_{SR}^{2}-h_{4}^{2}d_{3}^{m}\sigma_{PU_{2}}^{2}+P_{ST}h_{3}^{2}h_{4}^{2}}{2P_{ST}h_{3}^{2}h_{4}^{2}}$$

According to C7 and Equation (21), the following constraint can be obtained:

(22) $$\left|h_{3}^{2}d_{4}^{m}\sigma_{SR}^{2}-h_{4}^{2}d_{3}^{m}\sigma_{SR}^{2}\right|<P_{ST}h_{3}^{2}h_{4}^{2}$$

As $\left|h_{3}^{2}d_{4}^{m}\sigma_{SR}^{2}-h_{4}^{2}d_{3}^{m}\sigma_{SR}^{2}\right|$, $h_{3}^{2}$ and $h_{4}^{2}$ are non-negative values,$P_{ST}>0$ is always satisfied. By substituting Equations (3), (9) and (12) into Equation (18), the optimization problem of (18) can be described as follows:

(23) $$\begin{array}{lll} & P2: & \underset{P_{PU_{1}},\alpha ,\gamma }{\max}\;\alpha \log_{2}(1+\frac{(1-\beta )P_{PU_{1}}h_{1}^{2}}{d_{1}^{m}\sigma_{ST}^{2}})+(1-\alpha )\log_{2}(1+\frac{\gamma^{*}P_{ST}h_{3}^{2}}{d_{3}^{m}\sigma_{PU_{2}}^{2}})\\ & & +(1-\alpha )\log_{2}(1+\frac{(1-\gamma^{*})P_{ST}h_{3}^{2}}{d_{3}^{m}\sigma_{PU_{2}}^{2}})\\ s.t. & & C9:(1-\alpha )\log_{2}(1+\frac{\gamma^{*}P_{ST}h_{3}^{2}}{d_{3}^{m}\sigma_{PU_{2}}^{2}})>R_{t}\\ & & C10:(1-\alpha )\log_{2}(1+\frac{(1-\gamma^{*})P_{ST}h_{4}^{2}}{d_{4}^{m}\sigma_{SR}^{2}})>R_{t}\\ & & C11:\left|h_{3}^{2}d_{4}^{m}\sigma_{SR}^{2}-h_{4}^{2}d_{3}^{m}\sigma_{SR}^{2}\right|<P_{ST}\\ & & C12:0<\alpha <1,0<\beta <1\\ & & C13:P_{PU_{1}}>0\\ & & C1,C4,\;C5,C6 \end{array}$$

However, the objective function of P2 is still a non-convex function, and the feasible region is not a convex set. In order to obtain the global optimal solution, a slack variable $\omega =\alpha P_{PU_{1}}$ is introduced [29], and the optimization problem (Equation (23)) is rewritten as

(24) $$\begin{array}{lll} & P3: & \underset{\omega ,\alpha }{\max}\;\alpha \log_{2}(1+\frac{(1-\beta )\omega h_{1}^{2}}{\alpha d_{1}^{m}\sigma_{ST}^{2}})+(1-\alpha )\log_{2}(1+\frac{\gamma^{*}P_{ST}h_{3}^{2}}{d_{3}^{m}\sigma_{PU_{2}}^{2}})\;\\ & & \;\;\;\;\;+(1-\alpha )\log_{2}(1+\frac{\gamma^{*}P_{ST}h_{3}^{2}}{d_{3}^{m}\sigma_{PU_{2}}^{2}})\\ s.t. & & C14:\alpha \log_{2}(1+\frac{(1-\beta )\omega h_{1}^{2}}{\alpha d_{1}^{m}\sigma_{ST}^{2}})>R_{t}\\ & & C15:\frac{\alpha \beta \eta \omega h_{1}^{2}T}{\alpha d_{1}^{m}}>(1-\alpha )T*P_{ST}\\ & & C16:0<\frac{\omega }{\alpha }\leq P_{T\max}\\ & & C5,C9,\;C10,C11,C12 \end{array}$$

Now, the objective function of P3 is a concave function, and the feasible region is a convex set (Appendix C for the proof). However, due to the nature of the objective function and constraints, a joint closed-form solution cannot be obtained. Therefore, based on the idea of cross-layer optimization, this paper transforms the original optimization problem into an inner and outer two layers to solve.

Under the requirement of ensuring the normal communication of the system, the inner layer converts the optimization objective function into a subtractive formula. Then, the sub-gradient descent is used to obtain the optimal transmission power of $PU_{1}$. The outer layer obtains the optimal time switching factor α by unary linear optimization when the optimal transmission power of $PU_{1}$ has been obtained.

### 3.1. Power Control

This section is the inner layer of M-TPRA, solving the power control problem. The optimization variable is $P_{PU1}$. The Lagrange equation of the sub-problem can be described as

(25) $$\begin{array}{ll} P4: & \underset{\omega }{\max}-\alpha \log_{2}(1+\frac{(1-\beta )\omega h_{1}^{2}}{\alpha d_{1}^{m}\sigma_{ST}^{2}})-(1-\alpha )\log_{2}(1+\frac{\gamma^{*}P_{ST}h_{3}^{2}}{d_{3}^{m}\sigma_{PU_{2}}^{2}})\\ & \;\;-(1-\alpha )\log_{2}(1+\frac{(1-\gamma^{*})P_{ST}h_{3}^{2}}{d_{3}^{m}\sigma_{PU_{2}}^{2}})\\ & \;\;+\lambda_{1}(R_{t}-\alpha \log_{2}(1+\frac{(1-\beta )\omega h_{1}^{2}}{\alpha d_{1}^{m}\sigma_{ST}^{2}}))\\ & \;\;+\lambda_{2}((1-\alpha )T*P_{ST}-\frac{\alpha \beta \eta \omega h_{1}^{2}T}{\alpha d_{1}^{m}})\\ & \;\;+\lambda_{3}(\omega -\alpha P_{T\max})\\ & \;\;0<\alpha <1, \end{array}$$

where $\lambda_{i}(i=1,2,3)$ are dual variables. We apply Karush-Kuhn-Tucker (KKT) conditions and obtain

(26) $$\overset{⌢}{\omega }={\left[\frac{\left(1+\lambda_{1})\alpha (1-\beta )d_{1}^{m}h_{1}^{2}-\alpha d_{1}^{m}\sigma_{ST}^{2}(\lambda_{1}d_{1}^{m}-\lambda_{2}\beta \eta h_{1}^{2}\right)}{\left(1-\beta )h_{1}^{2}(\lambda_{1}d_{1}^{m}-\lambda_{2}\beta \eta h_{1}^{2}\right)}\right]}^{+}$$

(27) $${\overset{⌢}{P}}_{PU_{1}}=\frac{\overset{⌢}{\omega }}{\alpha }={\left[\frac{\left(1+\lambda_{1})(1-\beta )d_{1}^{m}h_{1}^{2}-d_{1}^{m}\sigma_{ST}^{2}(\lambda_{1}d_{1}^{m}-\lambda_{2}\beta \eta h_{1}^{2}\right)}{\left(1-\beta )h_{1}^{2}(\lambda_{1}d_{1}^{m}-\lambda_{2}\beta \eta h_{1}^{2}\right)}\right]}^{+}$$

${\left[x\right]}^{+}$ represents max{0,x}. From Equation (27), it can be known that ${\overset{⌢}{P}}_{PU_{1}}$ is independent of α. $\lambda_{i}(i=1,2,3)$ can be obtained by the sub-gradient method:

(28) $$\begin{array}{l} \lambda_{1}{}^{\left(k+1\right)}=\lambda_{1}{}^{\left(k\right)}+\xi (R_{t}-\alpha \log_{2}(1+\frac{(1-\beta )\omega h_{1}^{2}}{\alpha d_{1}^{m}\sigma_{ST}^{2}}))\\ \lambda_{2}{}^{{}^{\left(k+1\right)}}=\lambda_{2}{}^{\left(k\right)}+\xi ((1-\alpha )T*P_{ST}-\frac{\alpha \beta \eta \omega h_{1}^{2}T}{\alpha d_{1}^{m}})\\ \lambda_{3}{}^{{}^{\left(k+1\right)}}=\lambda_{3}{}^{\left(k\right)}+\xi (\omega -\alpha P_{T\max}) \end{array}$$

where *k* is the number of iterations and ξ is the negative gradient step size of γ in each iteration. After γ is updated, the value of $\overset{⌢}{\omega }$ is simultaneously updated. The specific implementation steps of the algorithm are shown in Algorithm 1.
**Algorithm 1:** Multi-User Time-Power Resource Allocation Algorithm1. Initialization: $P_{PU1}$, γ, $\lambda_{i}{}^{\left(k\right)}(i=1,2,3)$, $\Omega_{\alpha }$, convergence toleranceΔ; 2. while $\left|\lambda_{i}{}^{\left(k\right)}-\lambda_{i}{}^{\left(k-1\right)}\right|>\Delta (i=1,2,3)$ 3.    calculated $\gamma^{*}$, $P_{PU1}$ according to Equations (22) and (27); 4.    if $P_{PU1}>0$ and $P_{PU1}<P_{T\max}$ 5.     update ${\overset{⌢}{P}}_{PU1}$ 6.    end if 7.    calculated τ according to Equation (17); 8.    iteratively update $\lambda_{i}{}^{\left(k+1\right)}(i=1,2,3)$ according to Equation (29); 9. end while 10. obtained $\Omega_{\alpha }$ according to P5; 11. for $\alpha \in \Omega_{\alpha }$ 12.  calculated τ according to Equation (17); 13. end for

### 3.2. Time Allocation

This section is the outer layer of M-TPRA, solving the time allocation problem. That is, when the optimal transmission power of $PU_{1}$ is known, the maximum average throughput of the network is calculated. The sub-problems can be written as

(29) $$\begin{array}{lll} & P5: & \underset{\alpha }{\min}\;-\alpha \log_{2}(1+\frac{(1-\beta ){\overset{⌢}{P}}_{PU_{1}}h_{1}^{2}}{d_{1}^{m}\sigma_{ST}^{2}})\;-(1-\alpha )\log_{2}(1+\frac{\gamma^{*}P_{ST}h_{3}^{2}}{d_{3}^{m}\sigma_{PU_{2}}^{2}})\\ & & \;\;\;-(1-\alpha )\log_{2}(1+\frac{(1-\gamma^{*})P_{ST}h_{3}^{2}}{d_{3}^{m}\sigma_{PU_{2}}^{2}})\\ s.t. & & C17:\alpha \log_{2}(1+\frac{(1-\beta ){\overset{⌢}{P}}_{PU_{1}}h_{1}^{2}}{d_{1}^{m}\sigma_{ST}^{2}})>R_{t}\\ & & C18:\frac{\alpha \beta \eta h_{1}^{2}{\overset{⌢}{P}}_{PU_{1}}}{d_{1}^{m}}>(1-\alpha )*P_{ST}\\ & & C19:(1-\alpha )\log_{2}(1+\frac{\gamma^{*}P_{ST}h_{3}^{2}}{d_{3}^{m}\sigma_{PU_{2}}^{2}})>R_{t}\\ & & C20:(1-\alpha )\log_{2}(1+\frac{(1-\gamma^{*})P_{ST}h_{4}^{2}}{d_{4}^{m}\sigma_{SR}^{2}})>R_{t}\\ & & C21:0<\alpha <1 \end{array}$$

The optimization problem P5 is a unary linear optimization. The value range $\Omega_{\alpha }$ of α can be calculated through the constraints of P5. Then, the maximum value of the objective function can be obtained within this range. The solution detailed steps of the multi-user time-power resource allocation algorithm (M-TPRA) is shown in Algorithm 1.

## 4. Results and Discussion

### 4.1. Simulation Parameters

In this section, M-TPRA is simulated, verified and analyzed considering a pair of PUs and a pair of SUs. The part values of the simulation parameters were based on ref. [28] and are summarized in Table 1.

### 4.2. Effect of Transmission Power on Throughput

**Figure 3:**
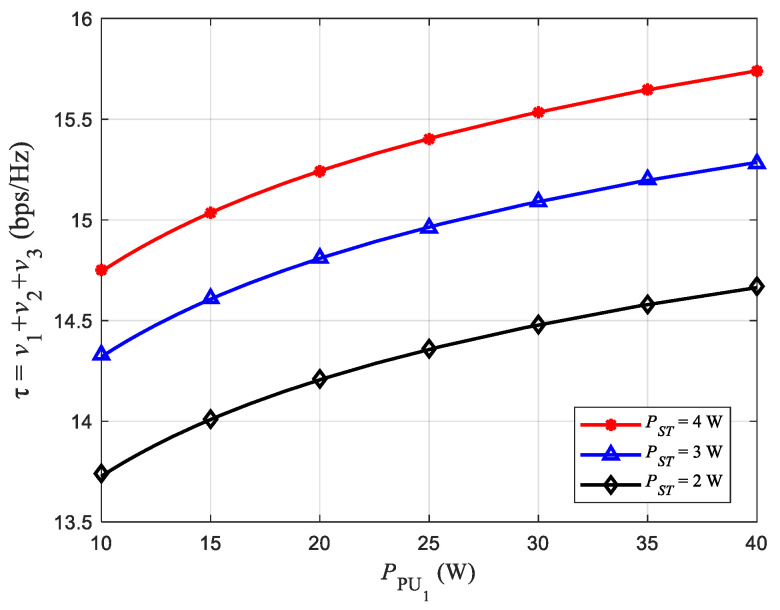
Effect of $P_{PU1}$ on average network throughput.

Figure 3 shows the relationship between transmission power $P_{\mathrm{PU}1}$ and the average throughput of the network, where the value of α is 0.5.

For horizontal comparison, Figure 3 shows the relationship between $P_{PU1}$ and the network average throughput. It can be seen from Figure 3 that as $P_{PU1}$ increases, the network average throughput increases gradually. This phenomenon is mainly caused by two reasons. Firstly, when the noise power is constant, the greater the transmission power, the greater the $SINR_{ST}$, the greater the maximum transmission rate $v_{1}$ on the communication link from $PU_{1}$ to ST. Hence, the network average throughput increases. Secondly, the network adopts the DF relay strategy. The larger $SINR_{ST}$ is, the probability of successful ST decoding will also increase, which is also conducive to improving the network average throughput. However, it is due to external interference and the limitation of $P_{T\max}$ that the network average throughput grows slower as $P_{PU1}$ becomes larger.

When comparing vertically, Figure 3 shows the relationship between the $P_{ST}$ and the network average throughput. Observing Figure 3, it can be seen that when $P_{PU1}$ is kept constant, the network average throughput increases with the increase of $P_{ST}$. The reason is similar to that in the horizontal comparison. However, with the increase of $P_{ST}$, the curve in Figure 3 gradually becomes denser, indicating that the average network throughput grows slowly, which is due to the influence of the interference threshold $I_{th}$.

### 4.3. Relationship between α and Throughput

**Figure 4:**
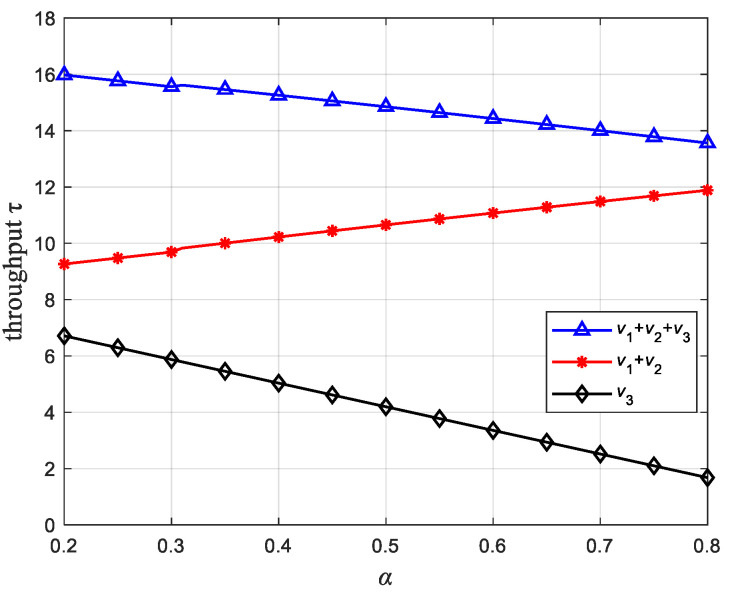
Relationship between α and throughput.

Figure 4 is the relationship between α and the network average throughput, where $P_{ST}=2W$. In the network, the PU network contains two communication links, $PU_{1}\to ST$ and $ST\to PU_{2}$, and the SU network contains one communication link, ST→SR, so the PU network throughput is $v_{1}+v_{2}$, the SU network throughput is $v_{3}$, and the average network throughput is $v_{1}+v_{2}+v_{3}$. First of all, we observe from Figure 4 that the network average throughput, the PU network throughput and the SU network throughput cannot be optimal at the same time. Secondly, when other parameters in the network are determined, α is linearly related to the average throughput of the network. As α increases, $v_{1}$ gradually becomes larger, so α has a positive linear relationship with the network throughput of PU. However, α has a negative linear correlation with the SU network throughput and the network average throughput. This is because the increase of α shortens the time left for ST to propagate information. The maximum transmittable rate on the $ST\to PU_{2}$, ST→SR two communication links becomes smaller, which may be lower than the target rate $R_{t}$, which leads to the interruption of the link. Thus, the throughput is reduced. In this system model, the goal is to maximize the network average throughput, so α should take the minimum value in the range $\Omega_{\alpha }$.

### 4.4. Effect of Distance on γ

**Figure 5:**
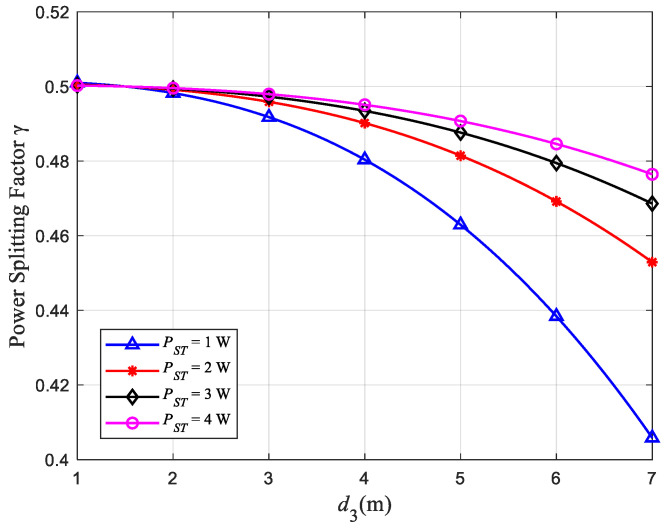
Relationship between $d_{3}$ and γ.

**Figure 6:**
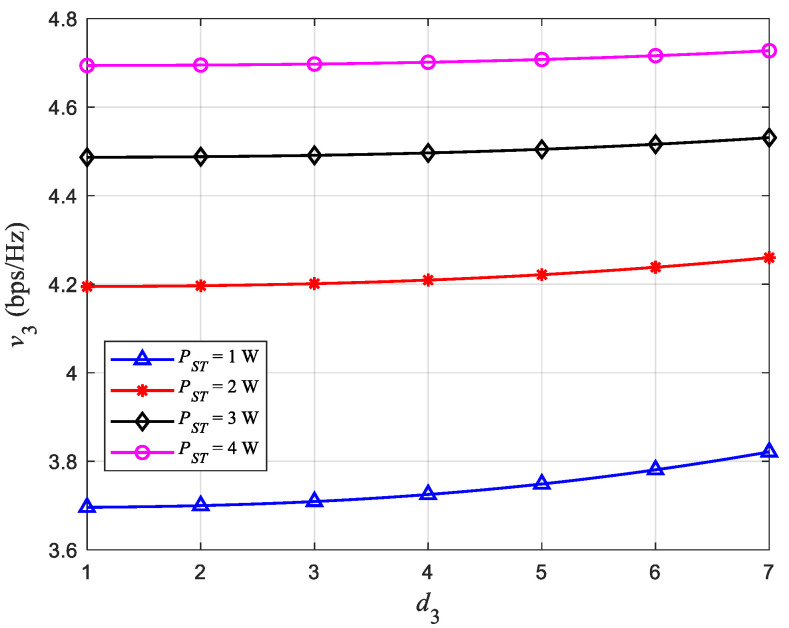
Relationship between $d_{3}$ and *v*_3_.

Figure 5 shows the relationship between the distance $d_{3}$ from ST to $PU_{2}$ and γ at ST, and Figure 6 shows the relationship between $d_{3}$ and *v*_3_. Among them, the distance $d_{4}$ from ST to SR remains unchanged at 1.5 m. When $d_{3}=d_{4}=1.5m$, it can be observed from Figure 5 that γ=0.5, that is, ST is always in the state of equal power distribution whatever the value of $P_{ST}$. In addition, when $P_{ST}$ remains unchanged, γ gradually decreases with the increase of $d_{3}$, which results in less transmission power allocated by ST to $PU_{2}$. This is because ST reserves more power for the SR to ensure the communication quality of the close-range users. Therefore, from Figure 6 we can observe that the throughput *v*_3_ of the SU network communication link ST→SR becomes larger as $d_{3}$ becomes larger. At the same time, when $d_{3}$ and $d_{4}$ are kept constant, the larger the $P_{ST}$, the smaller the proportion of occupied transmission power; so, we can observe that γ decreases with the increase of $P_{ST}$.

**Figure 7:**
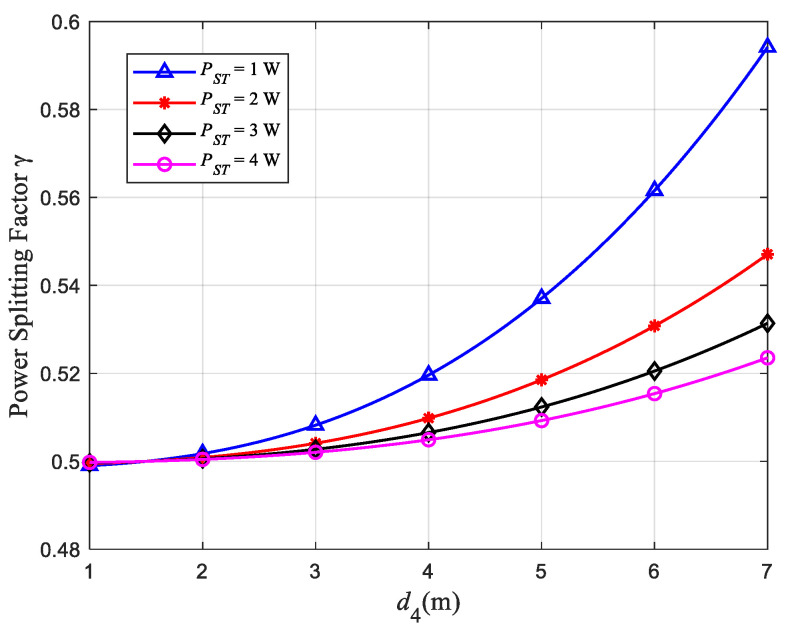
Relationship between $d_{4}$ and γ.

**Figure 8:**
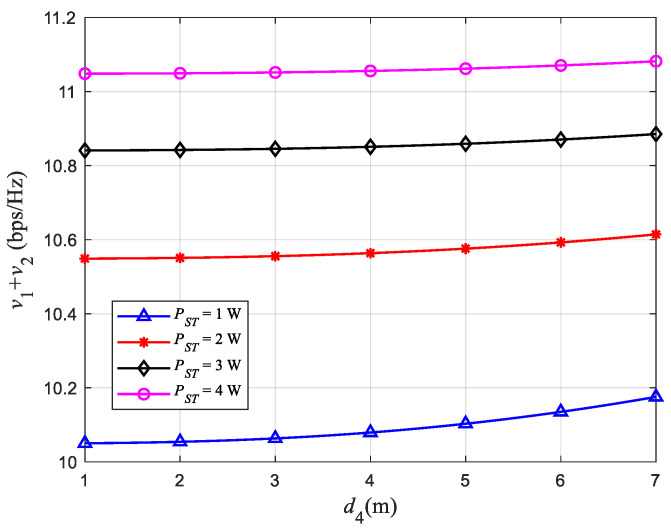
Relationship between $d_{4}$ and *v*_1_ *+ v*_2_.

Figure 7 is a relationship curve between the distance $d_{4}$ from ST to SR and γ. Figure 8 is a relationship curve between the distance $d_{4}$ and *v*_3_ *+ v*_2_. Among them, the distance $d_{3}$ from ST to $PU_{2}$ remains unchanged at 1.5 m. In the system model, the transmission power allocated by ST to SR is $(1-\gamma )P_{ST}$, which has a complementary relationship with the transmission power $\gamma P_{\mathrm{ST}}$ allocated to $PU_{2}$. Therefore, in Figure 7, the relationship $d_{4}$-γ and the relationship $d_{3}$-γ have opposite trends and are symmetrical about γ=0.5. The characteristics of the curves in Figure 7 are similar to those in Figure 5. At the same time, we can see from Figure 8 that the throughput of the PU network *v*_3_ *+ v*_2_ becomes larger as $d_{4}$ increases, which ensures the communication quality of the close-range user.

### 4.5. Algorithm Comparative Analysis

**Figure 9:**
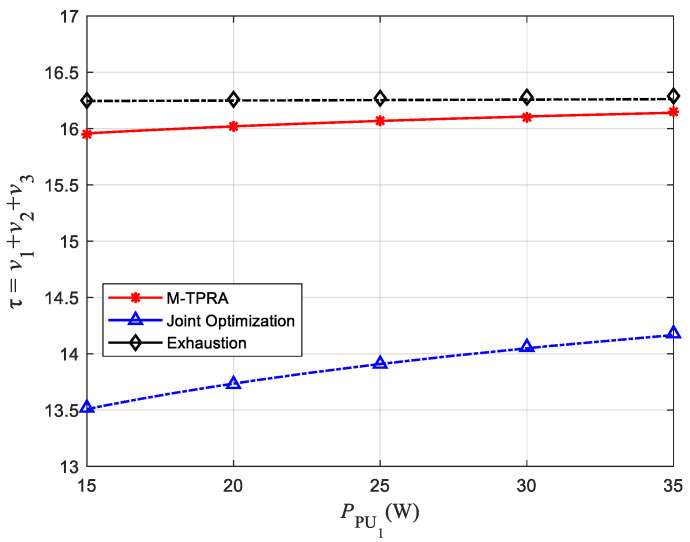
Performance comparison of M-TPRA, exhaustive method and joint optimization algorithm.

Figure 9 shows the performance comparison of M-TPRA, the exhaustive method and the joint optimization algorithm in this model. It can be seen from Figure 9 that with the increase of $P_{PU_{1}}$, the performance of M-TPRA gradually approaches the exhaustive method. However, the exhaustive method needs to traverse all possibilities, so it takes longer than M-TPRA. In the case of similar performance, M-TPRA is more applicable to the actual situation. The optimization goal of the joint optimization algorithm proposed in ref. [29] is end-to-end throughput. From Figure 9, it can be observed that the performance of M-TPRA is much higher than joint optimization. Therefore, the algorithm that optimizes the end-to-end throughput cannot guarantee the overall throughput of the network.

### 4.6. Energy Consumption Analysis

**Figure 10:**
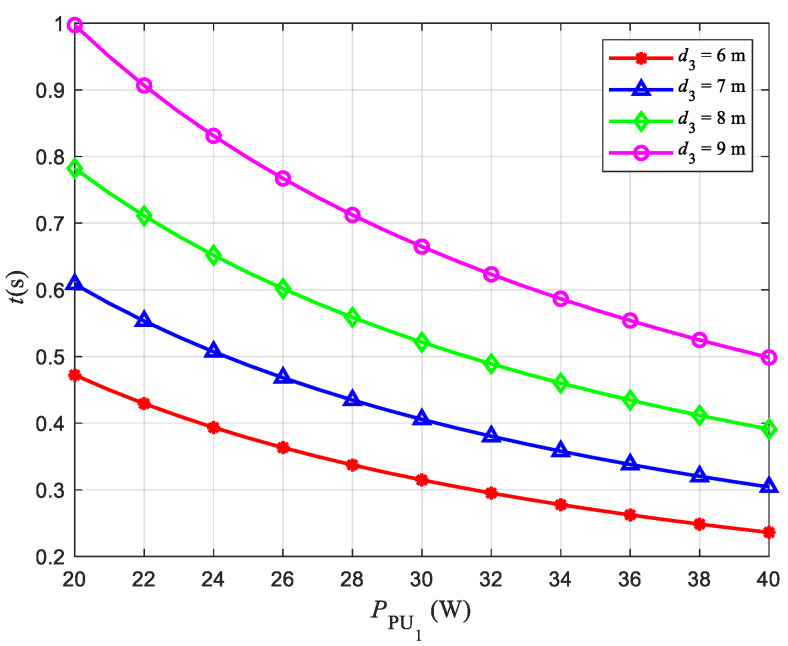
Relationship between $P_{PU_{1}}$ and the EH time.

In this system, we ignore the energy loss of the EH circuit when converting RF energy into electrical energy. Therefore, the energy used by the ST consists of only two parts, one for executing the M-TPRA algorithm and the other for transmitting information. In order to balance the communication quality of SR and $PU_{2}$, the maximum value of $d_{3}^{}$ is 9 m when $d_{4}^{}$ is 1.5 m. In addition, considering the saturation of the EH circuit, a timer is added to the EH system. The timer specifies the time required to collect energy. Figure 10 shows the relationship between the transmit power and the EH time when the distance between users is consistent. For example, when the value $P_{ST}$ is 20 W, the maximum energy collected by the system is 3.34 w. When the information transmission distance is 9 m, 2.64 W is required. Therefore, the energy consumed by executing the M-TPRA algorithm is about 0.7 W. Beyond this energy value, the communication link may be interrupted. The time required to collect energy $E_{ST}=\alpha \beta {\left|h_{1}\right|}^{2}P_{PU_{1}}T/d_{1}^{m}$ is αT, so the average speed of collecting energy is $e_{ST}=\beta {\left|h_{1}\right|}^{2}P_{PU_{1}}/d_{1}^{m}$. Therefore, when the energy required to execute the M-TPRA algorithm is 0.7 W, the time to collect 3.34 w energy is 1 s. It can be seen from Figure 10 that when the distance between users is fixed, the larger $P_{PU_{1}}$ is, the shorter the time required for energy collection is. When the transmit power is constant, the larger $d_{3}^{}$ is, the longer the energy collection time.

## 5. Conclusions

This paper studies the problem of maximizing average throughput in EH-CCRNs. In this network, SU relies on the energy collected from the primary user’s RF signal to decode and forward information, and the transmission power of both the PU and SU is limited to a certain extent. Under these constraints, a multi-user time-power resource allocation algorithm (M-TPRA) is proposed to improve the network average throughput. Simulation results show that the average throughput of the network is positively correlated with the primary users’ transmission power and negatively correlated with the system time switching factor. At the same time, the system can reasonably set the power splitting factor at the SU according to the user distance and give priority to ensuring the communication quality of the short-range users. In addition, the energy consumed by implementing the M-TPRA algorithm is also analyzed. In order to make the system more realistic, the saturation of the EH circuit is considered. A timer is added to the system, and the time set by the timer is related to the $P_{PU_{1}}$ and the distance between users. This work can also be considered in the scenario of multiple pairs of sub-users.

## Figures and Tables

**Table 1:** Simulation parameters.

Parameters	Value
energy conversion efficiency η	1
path loss index m	2.7
target rate $R_{t}/\mathrm{bps}/\mathrm{Hz}$	1
maximum transmission power $P_{\mathrm{Tmax}}/W$	50
power split factor β	0.5
interference threshold $I_{th}/W$	5
distance between users $d_{1}=d_{2}=d_{3}=d_{4}/m$	1.5
AWGN variance $\sigma_{\mathrm{ST}}^{2}=\sigma_{\mathrm{SR}}^{2}=\sigma_{\mathrm{PU}2}^{2}/W$	10^−3^

## Data Availability

Not applicable.

## Appendix A

**Table A1:** Notation list.

Notation	Description
$P_{PU_{1}}$	transmission power of $PU_{1}$
$P_{ST}$	transmission power of ST
$h_{k}(k=1,2,3,4)$	channel coefficient
$d_{k}(k=1,2,3,4)$	distance between users
$n_{ST}$	zero-mean additive white Gaussian noise at ST
$n_{PU2}$	zero-mean additive white Gaussian noise at $PU_{2}$
$n_{SR}$	zero-mean additive white Gaussian noise at SR
$\sigma_{ST}^{2}$	AWGN variance at ST
$\sigma_{PU_{2}}^{2}$	AWGN variance at $PU_{2}$
$\sigma_{SR}^{2}$	AWGN variance at SR
m	path loss index
α	time switching factor
β	power-splitting factor
γ	energy conversion efficiency
$I_{th}$	interference threshold
$P_{T\max}$	maximum transmission power
$v_{k}(k=1,2,3)$	maximum transmission rate
$R_{t}$	target rate
τ	average network throughput
$E_{ST}$	energy collected by ST

## Appendix B

The Hessian matrix $H_{1}$ of optimization problem P1 can be expressed as

(A1) $$H_{1}=\left[\begin{array}{lll} \frac{\partial \tau^{2}}{\partial^{2}P_{PU_{1}}} & \frac{\partial \tau^{2}}{\partial P_{PU_{1}}\partial \alpha } & \frac{\partial \tau^{2}}{\partial P_{PU_{1}}\partial \gamma }\\ \frac{\partial \tau^{2}}{\partial \alpha \partial P_{PU_{1}}} & \frac{\partial \tau^{2}}{\partial^{2}\alpha } & \frac{\partial \tau^{2}}{\partial \alpha \partial \gamma }\\ \frac{\partial \tau^{2}}{\partial \gamma \partial P_{PU_{1}}} & \frac{\partial \tau^{2}}{\partial \gamma \partial \alpha } & \frac{\partial \tau^{2}}{\partial^{2}\gamma } \end{array}\right]$$

Bringing in the objective function of P1, we obtain

(A2) $$\left[\begin{array}{ccc} a_{11} & a_{12} & 0\\ a_{21} & 0 & a_{23}\\ 0 & a_{32} & a_{33} \end{array}\right]$$

where:

(A3) $$\begin{array}{l} a_{11}=-\frac{1}{\ln2}\frac{\alpha {\left(1-\beta \right)}^{2}h_{1}^{4}}{{\left(d_{1}^{m}\sigma_{ST}^{2}+(1-\beta )P_{PU_{1}}h_{1}^{2}\right)}^{2}}\\ a_{12}=\frac{1}{\ln2}\frac{(1-\beta )h_{1}^{2}}{d_{1}^{m}\sigma_{ST}^{2}+(1-\beta )P_{PU_{1}}h_{1}^{2}}\\ a_{21}=\frac{1}{\ln2}\frac{(1-\beta )h_{1}^{2}}{d_{1}^{m}\sigma_{ST}^{2}+(1-\beta )P_{PU_{1}}h_{1}^{2}}\\ a_{23}=-\frac{1}{\ln2}\frac{P_{ST}h_{3}^{2}}{d_{3}^{m}\sigma_{PU_{2}}^{2}+\gamma P_{ST}h_{3}^{2}}+\frac{P_{ST}h_{4}^{2}}{d_{4}^{m}\sigma_{SR}^{2}+(1-\gamma )P_{ST}h_{4}^{2}}\\ a_{32}=-\frac{1}{\ln2}\frac{P_{ST}h_{3}^{2}}{d_{3}^{m}\sigma_{PU_{2}}^{2}+\gamma P_{ST}h_{3}^{2}}+\frac{P_{ST}h_{4}^{2}}{d_{4}^{m}\sigma_{SR}^{2}+(1-\gamma )P_{ST}h_{4}^{2}}\\ a_{33}=-\frac{1}{\ln2}\frac{(1-\alpha )P_{ST}^{2}h_{3}^{2}}{{\left(d_{3}^{m}\sigma_{PU_{2}}^{2}+\gamma P_{ST}h_{3}^{2}\right)}^{2}}-\frac{(1-\alpha )P_{ST}^{2}h_{4}^{4}}{{\left(d_{4}^{m}\sigma_{SR}^{2}+(1-\gamma )P_{ST}h_{4}^{2}\right)}^{2}}\; \end{array}$$

Applying row operations, we obtain

(A4) $$\left[\begin{array}{ccc} b_{11} & b_{12} & 0\\ 0 & b_{22} & b_{23}\\ 0 & 0 & b_{33} \end{array}\right]$$

where:

(A5) $$\begin{array}{l} b_{11}=-\frac{1}{\ln2}\frac{\alpha {\left(1-\beta \right)}^{2}h_{1}^{4}}{{\left(d_{1}^{m}\sigma_{ST}^{2}+(1-\beta )P_{PU_{1}}h_{1}^{2}\right)}^{2}}\\ b_{12}=\frac{1}{\ln2}\frac{(1-\beta )h_{1}^{2}}{d_{1}^{m}\sigma_{ST}^{2}+(1-\beta )P_{PU_{1}}h_{1}^{2}}\\ b_{22}=\frac{1}{\ln2}\frac{1}{\alpha }\\ b_{23}=-\frac{1}{\ln2}\frac{P_{ST}h_{3}^{2}}{d_{3}^{m}\sigma_{PU_{2}}^{2}+\gamma P_{ST}h_{3}^{2}}+\frac{P_{ST}h_{4}^{2}}{d_{4}^{m}\sigma_{SR}^{2}+(1-\gamma )P_{ST}h_{4}^{2}}\\ b_{33}=-\frac{1}{\ln2}\frac{\alpha (1-\alpha )P_{ST}^{2}h_{3}^{2}}{{\left(d_{3}^{m}\sigma_{PU_{2}}^{2}+\gamma P_{ST}h_{3}^{2}\right)}^{2}}-\frac{\alpha (1-\alpha )P_{ST}^{2}h_{4}^{2}}{{\left(d_{4}^{m}\sigma_{SR}^{2}+(1-\gamma )P_{ST}h_{4}^{2}\right)}^{2}}\; \end{array}$$

It can be seen from the above equation that the eigenvalues of the Hessian matrix are $b_{11}$, $b_{22}$, and $b_{33}$, which are not all negative, so the objective function is not a convex function. In the same way, it can be seen that the constraint C1 is non-convex, while the constraints C2, C3, C4, C5, and C6 are convex, and the constraints C7 and C8 are affine surfaces, which are always convex.

## Appendix C

The Hessian matrix $H_{2}$ of optimization problem P3 can be expressed as

(A6) $$H_{2}=\left[\begin{array}{cc} \frac{\partial \tau^{2}}{\partial^{2}P_{PU_{1}}} & \frac{\partial \tau^{2}}{\partial P_{PU_{1}}\partial \alpha }\\ \frac{\partial \tau^{2}}{\partial \alpha \partial P_{PU_{1}}} & \frac{\partial \tau^{2}}{\partial^{2}\alpha }\; \end{array}\right]$$

Bringing in the objective function of P3, we obtain

(A7) $$\left[\begin{array}{cc} e_{11} & e_{12}\\ e_{21} & e_{22} \end{array}\right]$$

where:

(A8) $$\begin{array}{l} e_{11}=-\frac{1}{\ln2}\frac{\alpha {\left(1-\beta \right)}^{2}h_{1}^{4}}{{\left(\alpha d_{1}^{m}\sigma_{ST}^{2}+(1-\beta )\omega h_{1}^{2}\right)}^{2}}\\ e_{12}=\frac{1}{\ln2}\frac{{\left(1-\beta \right)}^{2}h_{1}^{4}\omega }{{\left(\alpha d_{1}^{m}\sigma_{ST}^{2}+(1-\beta )\omega h_{1}^{2}\right)}^{2}}\\ e_{21}=\frac{1}{\ln2}\frac{{\left(1-\beta \right)}^{2}h_{1}^{4}\omega }{{\left(\alpha d_{1}^{m}\sigma_{ST}^{2}+(1-\beta )\omega h_{1}^{2}\right)}^{2}}\\ e_{22}=-\frac{1}{\ln2}\frac{1}{\alpha }\frac{{\left(1-\beta \right)}^{2}h_{1}^{4}\omega^{2}}{{\left(\alpha d_{1}^{m}\sigma_{ST}^{2}+(1-\beta )\omega h_{1}^{2}\right)}^{2}} \end{array}$$

After row transformation, we obtain

(A9) $$\left[\begin{array}{cc} h_{11} & h_{12}\\ 0 & 0 \end{array}\right]$$

where:

(A10) $$\begin{array}{l} h_{11}=-\frac{1}{\ln2}\frac{\alpha {\left(1-\beta \right)}^{2}h_{1}^{4}}{{\left(\alpha d_{1}^{m}\sigma_{ST}^{2}+(1-\beta )\omega h_{1}^{2}\right)}^{2}}\\ h_{12}=\frac{1}{\ln2}\frac{{\left(1-\beta \right)}^{2}h_{1}^{4}\omega }{{\left(\alpha d_{1}^{m}\sigma_{ST}^{2}+(1-\beta )\omega h_{1}^{2}\right)}^{2}} \end{array}$$

The eigenvalues of the Hessian matrix are $h_{11}$ and 0, which are not positive, so the objective function is a concave function. Similarly, the eigenvalues of the constraints in P3 are all non-negative, so the feasible region of P3 is a convex set.
